# An inter-laboratory study of the multi-dimensional behaviors of analogue lumbar spine surrogates: towards standardization in spine testing

**DOI:** 10.3389/fbioe.2026.1769107

**Published:** 2026-04-09

**Authors:** Emma C. Coltoff, Siril Teja Dukkipati, Philip J. Brown, Mark Driscoll

**Affiliations:** 1 Department of Biomedical Engineering, Wake Forest School of Medicine, Winston-Salem, NC, United States; 2 Musculoskeletal Biomechanics Research Lab, Department of Mechanical Engineering, McGill University, Montréal, QC, Canada; 3 Orthopaedic Research Laboratory, Research Institute MUHC, Montreal General Hospital, Montréal, QC, Canada

**Keywords:** 3D printing, analogue, biomechanics, interlaboratory, lumbar, spine, surrogate

## Abstract

**Introduction:**

Experimental reproduction of lumbar spinal loading throughout activities of daily living is challenging and has historically relied on simplifications to the applied loading to pure moment testing along primary orthogonal anatomical planes. The primary objective of this study was to evaluate the reproducibility and crossplatform translation of multi-dimensional spinal loading protocols between two distinct biomechanical testing systems.

**Methods:**

Lumbar spinal surrogate models are used as repeatable, transportable, and inexpensive specimens to serve as standardized test articles for inter-laboratory comparison. Combined displacement-controlled Flexion-Extension (FE) and Lateral Bending (LB) trajectories and combined Axial Loading and Axial Rotation (AR) trajectories were applied to the specimens on a six-axis robotic arm and a two-axis linear-torsion testing system with custom jig. Both protocols were able to be decomposed and translated between the two testing systems, lending credibility to this method as a standardizable, reproducible process among biomechanics laboratories.

**Results:**

The results demonstrated strong agreement in combined FE-LB range of motion (ROM) and load across the two laboratory setups, with inter-laboratory ROM differences of 0.53°–0.55° (8%–10%) and peak load differences of 0.92–1.64 Nm (10%–18%) for the 3D printed surrogates. Combined AR and axial loading yielded similar behavior, with ROM differences of 0.80°–0.85° (3%–4%) and peak load differences of 0.21–0.89 Nm (1%–5%) between laboratories (all p > 0.05). Furthermore, both laboratories were able to utilize adopted methods to highlight specimen asymmetries and biomechanical responses that would be otherwise overlooked by traditional pure moment testing.

**Discussion:**

This work lays a foundation for future multidimensional testing for capturing complex biomechanical behavior of the spine.

## 1 Introduction

Surrogate models of the lumbar spine have been developed as standardized tools and as substitutes for cadaveric specimens, the current gold standard for biomechanical testing (Domann et al., 2011; Avidano et al., 2008). These surrogates are designed to improve reproducibility across implant testing and between different laboratories, facilitate long-term assessments that are difficult to perform using biological tissue, and mitigate challenges posed by logistics of specimen shipping and ethical approvals (Wheeler et al., 2011). Thus, the repeatability, transportability, and durability of these models make them ideally suited for experimental testing for the purposes of methodology development.

Validation of spinal surrogate models has historically been conducted through pure moment experimental loading, evaluating the relationship between the spine’s kinematic range of motion (ROM) in response to an applied moment along anatomical planes, i.e., Flexion-Extension (FE) in the sagittal plane, Lateral Bending (LB) in the coronal plane, and Axial Rotation (AR) in the transverse plane. However, human spinal motion in daily life often involves simultaneous combinations of FE, LB, and AR loading, making the simplification of their analysis to individual anatomical planes a potentially incomplete approach for characterization of spinal biomechanics (Holsgrove et al., 2015; Fuller et al., 2012; Cholewicki et al., 1996). This simplification may limit clinical translatability, as many pathological conditions and implant failure modes including asymmetric disc degeneration, facet joint overloading, pedicle screw loosening, and adjacent segment disease occur under combined and off-axis loading rather than isolated planar moments. For example, pure moment testing can underestimate facet and intradiscal stresses compared with more physiologically realistic load conditions that combine compression and bending (Rohlmann et al., 2009). Despite this evidence of the spine’s biomechanics as multiplanar *in situ*, over 70% of experimental biomechanical characterizations of passive spinal behavior and stability in literature are conducted in pure moment testing exclusively (Costi et al., 2021). Some notable deviations from the pure moment paradigm include simultaneous application of a bending moment and an axial compressive force (follower load) hypothesized to replicate the stabilizing effect of spinal muscle activation (Gardner-Morse and Stokes, 2004; Tushak et al., 2023). It is important to note that this constant, curvature-following compressive preload differs from the variable axial loading applied in the present study, in which compressive or tensile forces between the specimen mounting points are actively modulated during motion to interrogate load-dependent mechanical behavior of the spinal construct. Others have employed simultaneous linear ramping or sinusoidal combinations of FE/LB/AR (Schmidt et al., 2007; Schmidt et al., 2008; Spenciner et al., 2006; Zengerle et al., 2021).

While pure moment testing leaves the 90° sector between the FE (sagittal) and LB (coronal) planes untested, these studies have begun to close this gap by testing multiplanar loading combinations along planes that are 30° or 45° from pure moment bending axes with combinations of FE and LB or FE, LB, and AR (Daniels et al., 2012; Spenciner et al., 2006). However, the advent of industrial six-axis robots as well as enhancements to linear-torsion systems with custom jigs enable testing along planes of smaller increments than this, providing more resolution in understanding off-axis spinal behavior (Coltoff et al., 2025a). A multidimensional testing methodology introduced by Coltoff et al. leverages this opportunity by applying combinations of FE and LB loading in infinitesimal increments, thereby mapping spinal behavior between the neutral zone (NZ) and elastic zone (EZ) along previously untested planes (Coltoff et al., 2025a). The logic underlying this methodology can also be extended to a combination of axial loading and AR, as an analysis of coupled rotational and compressive/tensile loads on the lumbar spine.

For this study, two multiplanar loading combinations were chosen as testing modalities to represent physiologically relevant spinal motions: 1) a combination of FE and LB, and 2) a combination of AR and axial loading. Coupled sagittal and coronal plane bending has been widely documented in literature as an inherent feature of lumbar spine kinematics (Dukkipati and Driscoll, 2025a; Dukkipati and Driscoll, 2025c; Liebsch and Wilke, 2025; Cholewicki et al., 1996). Functionally, such coupled FE–LB motions occur during coordinated trunk movements involving rhythmic side-to-side trunk movements (e.g., hula-hooping). Similarly, the combination of AR and axial loading was selected based on evidence that compressive preload substantially influences rotational stiffness, neutral zone behavior, and segmental stability of the spine (Gardner-Morse and Stokes, 2004). *In vivo*, trunk rotation rarely occurs in the absence of compressive loading generated by body weight and muscle activation. Functionally, this loading scenario approximates motions such as seated trunk rotation while reaching posteriorly (e.g., retrieving an object from the back seat of a vehicle). Although many such combinations can be synthesized, care must be taken to preserve experimental practicality when decomposing these combined motions into the orthogonal loads, particularly when using two-axis linear-torsion systems.

Building on this rationale, the objectives of this work were threefold: 1) to assess performance of two physiologically-inspired combined loading protocols using standardized surrogate models, 2) to develop a standardized procedure for implementing these combined loading protocols, and 3) to compare the outcomes from these combined loading protocols across two distinct testing systems.

## 2 Methods

### 2.1 Spinal surrogate models

**FIGURE 1:**
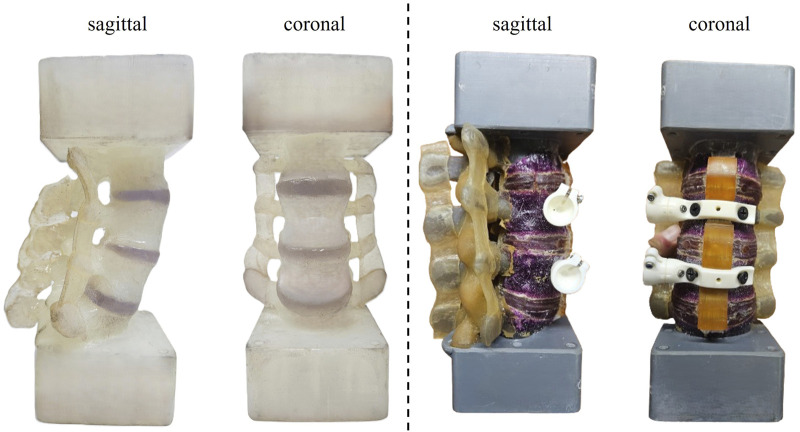
L2-L5 lumbar spine surrogates tested: (left) 3D-printed and (right) Sawbones composite surrogate.

Two L2-L5 lumbar spine surrogate models were utilized for the work in this study: a novel validated 3D-printed surrogate model (n = 3) (Dukkipati and Driscoll, 2024; Dukkipati and Driscoll, 2025b) and a commercially available biomechanical model (Sawbones®, WA, United States, SKU# 3430-25 (4), n = 1) (Zdero et al., 2023) (Figure 1). This investigation was designed as a preliminary proof-of-concept study to assess the mechanical feasibility and inter-laboratory reproducibility of multiplanar loading protocols in standardized surrogate constructs, rather than to establish population-level statistical inference.

The 3D printed surrogate was developed by Dukkipati and Driscoll in the Musculoskeletal Biomechanics Research Lab at McGill University (Dukkipati and Driscoll, 2024; Dukkipati and Driscoll, 2025b). It consists of L2 to L5 solid vertebral bodies and their corresponding intervertebral discs (IVDs)– the fibrocartilaginous cushion between vertebrae – which are derived from the open-source human anatomography database “BodyParts3D” (Mitsuhashi et al., 2009) and the manually designed interspinous and the intertransverse ligaments using the anatomical landmarks on the spinous processes between the adjacent vertebrae. The source dataset corresponds to a healthy 22-year-old male (height: 172.8 cm; mass: 65.0 kg). No geometric scaling was applied during model preparation. The resulting construct demonstrated a lumbar lordosis angle of 44.76°, which aligns closely with reported normative values for asymptomatic young adults (47° ± 10.07°) (Kim et al., 2003). Each anatomical component was printed separately on a Form 3 L printer with default settings at a 100 µm layer height (Formlabs Inc., MA, United States) and assembled into a functional spine unit (FSU). The vertebrae were printed in Formlabs Durable v2 resin and the IVDs and ligaments in Formlabs Flexible 80A V1 photopolymer resin. Post-processing steps followed manufacturer recommendations and involved washing the components in 99% isopropyl alcohol solution to remove excess resin and curing under a 405 nm ultraviolet light source. The parts were then bonded together by applying a small quantity of Flexible 80A v1 resin between the surfaces and spot curing them. After all the vertebral levels were assembled using a jig, the whole model was then cured for 60 min in UV light to ensure proper bonding. Three identical versions of this model were created for this study. The L1-S1 versions of these models were validated previously against literature *ex vivo* (Stubbs, 2011; Rao, 2012) and *in silico* (Campbell et al., 2016; Rao, 2012) datasets in pure moment.

The construction of the Sawbones model included proprietary composite materials for each of the vertebrae, IVDs, and interspinous ligaments, with grey resin mounting blocks above L2 and below L5. The geometry of these blocks was integrated into the design of the 3D printed surrogate such that the same mounting adapter could be used for both models. Both the 3D printed model and the Sawbones model were dimensionally similar, with the features of the 3D printed model between the blocks being 1 cm shorter than the Sawbones model in the cranio-caudal direction. The comparisons in this study are limited to construct-level mechanical responses rather than intrinsic material equivalence.

### 2.2 Testing environments

Two laboratories with different testing equipment were employed in this study to compare the repeatability of the multiplanar loading methodology across testing systems. These setups enabled simultaneous FE and LB bending, as well as combined AR bending and axial loading.

**FIGURE 2:**
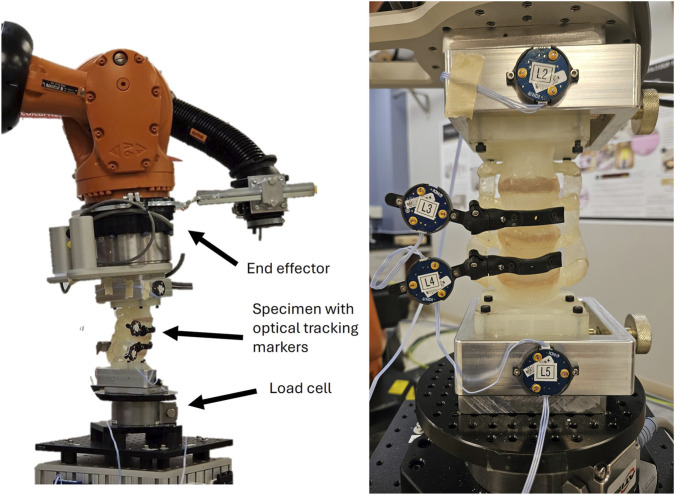
Testing setup for laboratory with six-axis robotic arm. (left) 3D printed specimen on robot, (right) close-up of the specimen with optical tracking sensors.

In one setup (Wake Forest University), an industrial six-axis robotic arm, KR 300 R2500 Ultra Robot (KUKA Robotics, Augsburg, Germany), is utilized for biomechanical testing (Figure 2). Each L2-L5 surrogate was mounted to custom fixtures. The fixture at the caudal end of the specimen was mounted to a Delta IP68 SI-330-30 six-axis (ATI Industrial Automation Inc., United States) load cell atop a stand rigidly affixed to the floor. The fixture at the cranial end was mounted to the end effector of the KUKA system. Geometrically similar mounting adapters were used across both laboratories to define the construct’s cranio-caudal axis and ensure consistent boundary conditions. Optotrak (Northern Digital Inc., Canada) optical tracking sensors were affixed to each the L3 and L4 vertebral bodies via custom 3D-printed sensor mounts. One sensor each was attached to the metal fixtures at the cranial and caudal ends of the specimen for L2 and L5. simVITRO (Cleveland Clinic BioRobotics, United States) software was used to interpret the specimen’s ROM behavior from the optical tracking sensors and load behavior from the load cell. Four landmark points were digitized for each of the lumbar vertebra to set up a rigid body coordinate system for the vertebral bodies; the L3-L4 FSU was selected as the central location of the joint coordinate system (JCS) on the specimen for simVITRO’s gravity compensation procedure (simVITRO bioRobotics, 2014). Utilizing the defined rigid body coordinate systems, sensor translations and rotations from the Optotrak system were transformed through simVITRO into anatomical bending measures in anterior, lateral, and superior translations (mm) and FE, LB, and AR bending (degrees) relative to the JCS for the segment and individual FSUs of the spine. The ATI load cell measures were processed through simVITRO to determine the anterior, lateral, and superior forces (N) and FE, LB, and AR bending moments (Nm) on the spine segment in response to loading. Pure moments were achieved through active six-degree-of-freedom control using real-time load cell feedback to minimize off-axis forces and moments during testing.

**FIGURE 3:**
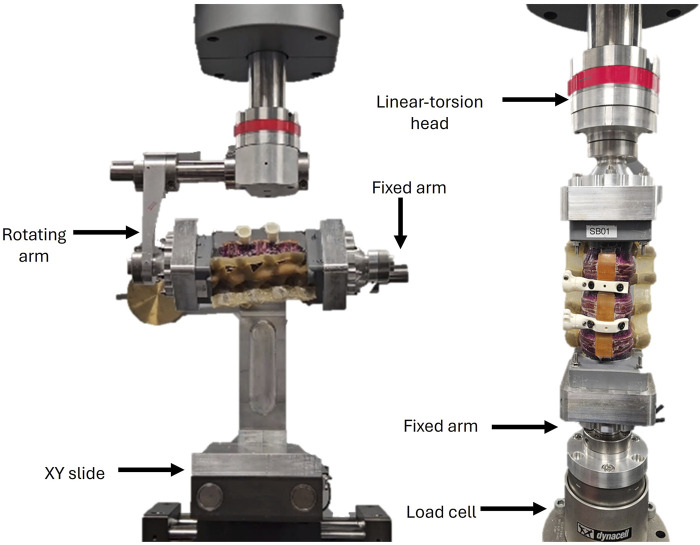
Testing setups on two-axis linear-torsion testing system setups with the Sawbones specimen for (left) FE and LB, and (right) AR and axial loading.

In the second setup (McGill University), a two-axis linear-torsion testing machine (Electroplus E10000, Instron, MA, United States) was used along with a custom bending jig (Dukkipati and Driscoll, 2024; Dukkipati and Driscoll, 2025b) to enable simultaneous orthogonal linear and rotary load application (Figure 3). The jig consisted of a rotating arm attached to the mounting adapter of L2 vertebra, and a fixed arm attached to the adapter of the L5 vertebra. The adapters allow for rotation of the specimen along its longitudinal axis, making it possible along the sagittal plane, coronal plane, and any plane in between. The rotational axis of the jig was mechanically aligned with the longitudinal midline of the mounting adapters to maintain consistency with the construct axis defined in the robotic setup. A free-moving XY slide ensured no off-axis load in two of the three translational degrees of freedom, while the third translational direction was passively controlled by the linear-torsion head. This XY slide permitted dynamic translation of the specimen during loading, allowing it to self-equilibrate under applied moments and thereby minimizing shear forces and eccentric loading. The XY slide sits on a bi-axial load cell (Dynacell™ 2527-202, Instron, MA, United States, range ±10 kN and ±100 Nm, resolution 0.1 N and 0.05 Nm) measuring the moment and force imparted onto the specimen during testing. Off-axis loads were monitored via the load cell and remained negligible during testing (<5N), consistent with prior validation of this setup (Dukkipati and Driscoll, 2025b; Dukkipati and Driscoll, 2024).

### 2.3 Loading protocols

Previous work has validated each of the spinal surrogates through pure moment loading protocols (DiAngelo et al., 2019; Domann et al., 2011; Dukkipati and Driscoll, 2024; Dukkipati and Driscoll, 2025b; Campbell et al., 2012). These studies were used as a reference for designing the multiplanar loading protocol in this work. Two distinct combinations of loads, first described in (Coltoff et al., 2025b), were applied to the surrogate models: FE and LB and AR and axial loading. Both loading schemes were designed using moment-controlled ROM boundaries determined by testing preceding combined loading. In all loading schemes, the cranial end of the specimen was rotated while the caudal end was fixed.

#### 2.3.1 Moment-controlled boundary

**FIGURE 4:**
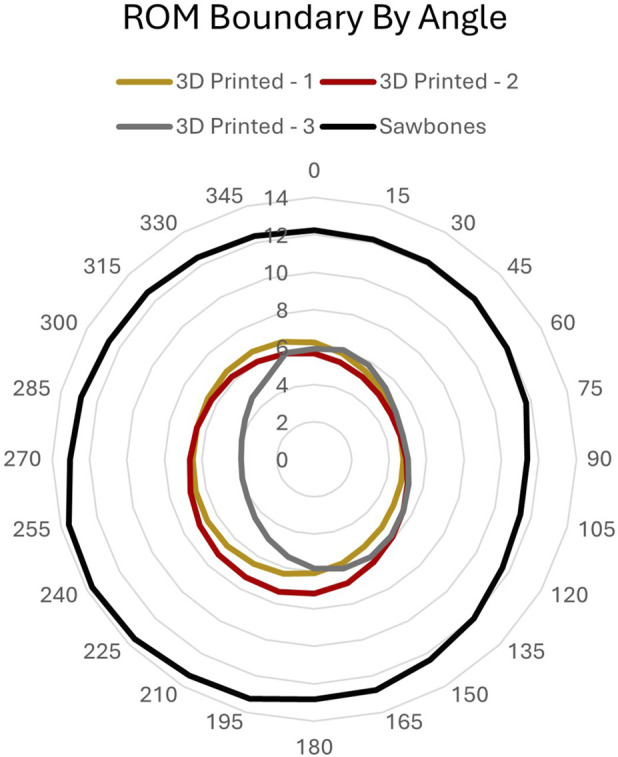
FE-LB ROM boundaries defined by six-axis robotic arm.

For combined FE and LB, a “boundary” of combined FE and LB loads of 7.5 Nm (Costi et al., 2021) was applied 360° about the craniocaudal axis under a moment-controlled loading scheme to capture the ROM boundary of each specimen corresponding to the 7.5 Nm load (Figure 4). This 7.5 Nm safety limit was chosen to reflect the validity of use of the 3D printed models in previous validation studies (Dukkipati and Driscoll, 2024; Dukkipati and Driscoll, 2025b).

For combined AR and axial loading, a boundary for AR rotations was obtained by pure moment loading of the specimen via a sinusoidal load up to an amplitude of 7.5 Nm at a rate of approximately 0.3°/second.

#### 2.3.2 Combined flexion-extension and lateral bending

**FIGURE 5:**
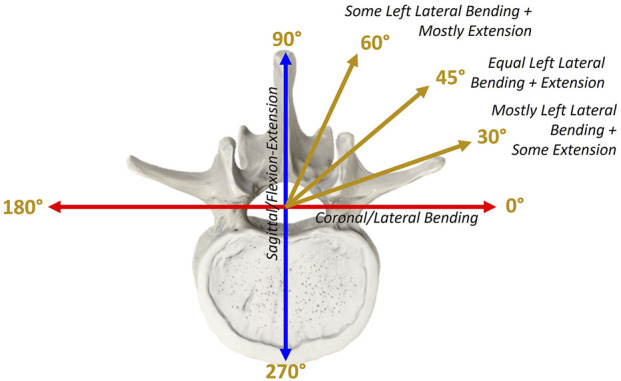
Polar coordinate system definition relative to craniocaudal axis.

The FE-LB boundary was used to define a trajectory of combined FE and LB rotations, known as “rays”, originating from the origin (no-load position) and ending at the ROM boundary in a straight line. A hybrid control scheme, where forces/translations in load-control and moments/rotations where displacement-control was used for this. The rays trajectory approximates standard pure moment bending wherein the spine moves from the neutral zone (NZ) through the elastic zone (EZ) to the end of the spine’s ROM along the pre-defined boundary, then returns back through the NZ to the end ROM in the direction of the explementary angle. This loading is applied along planes defined by steps of 5° about the craniocaudal axis, where the zero-degree axis is at pure Extension (Figure 5). Specimen ROM and loading in 6 DOF were recorded in response to the rays trajectory, with particular interest in FE and LB moments.

**FIGURE 6:**
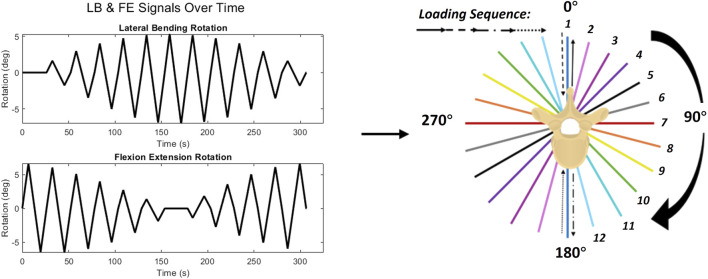
(left) Combined FE and LB trajectory to simulate rays, (right) rays trajectory decomposed into loading planes, with experimental sequence annotated.

To employ the rays trajectory on the two-axis linear-torsion testing machine, the trajectory must be decomposed along individual loading planes relative to the NZ. Individual planes are denoted by a numeric identifier from 1-12 in Figure 6 by their primary angle, and their explementary angle is found by following the line through the NZ. The ROM boundary of each specimen corresponding to 7.5 Nm of moment in each plane was first recorded using the six-axis robotic arm. These points were used to define the displacement-controlled trajectories applied by the uniaxial testing machine via a linear ramp between the end points with a prescribed loading rate of 1°/sec. Loading along each plane began from a neutral position of the spine where all forces and moments on the segment were minimized. Loading was then applied towards extension, ending at the boundary, then back through the neutral position towards flexion, ending of the boundary, returning to the neutral position.

The specimen was manually rotated 15° (error of ±0.5°), verified by a digital angle gauge (Klein Tools, IL, United States) after each loading cycle to align with the appropriate loading planes until loading along all 12 planes was completed. Specimens were not removed from the fixture until loading in all planes was completed.

#### 2.3.3 Combined axial rotation and axial loading

**FIGURE 7:**
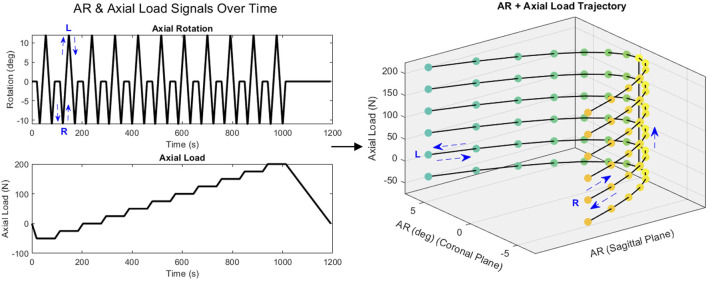
AR sweeping pattern with stepwise ramping of axial load.

**FIGURE 8:**
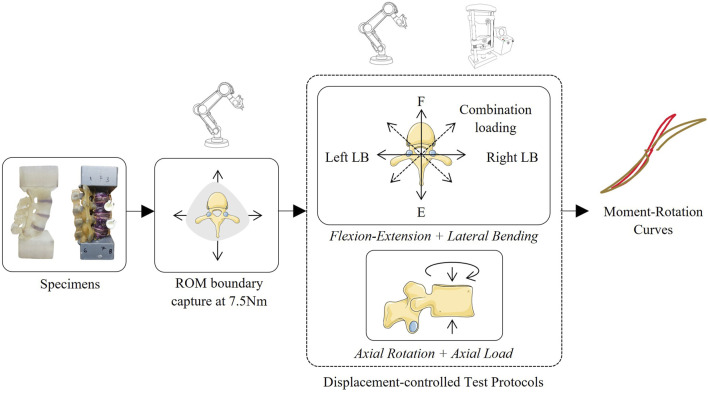
Summary of the testing and analysis methodology in this study.

The resulting rotations from the AR boundary capture were used to design a linear ramping signal to capture the specimen’s behaviors approaching and crossing through the neutral position from both AR end points. The control signal sweeps from the neutral position, to the end point on the boundary in right AR, back through the neutral position to the end point in left AR, reversing direction back through the neutral position to the end point in right AR again, and finally returning to the neutral position. For the combined loading, this pattern was applied in tandem with an axial load at 11 loading levels, in increments of 25 N from 50 N of tension (−50 N) to 200 N of compression (Figure 7). Specimen loading and ROM in 6 DOF were recorded in response to the rays trajectory, with particular interest in AR moments and superior translations. A schematic representation of both these protocols is provided in Figure 8.

### 2.4 Post-processing

After testing, 6 DOF ROM and load for the L2-L5 spinal segment were transformed into resultant ROM, load, and stiffness values. The data are presented as 3D representations of these values as well as 2D load-displacement responses along the loading planes (for FE and LB combined loading) through custom MATLAB (Mathworks, California, United States) scripts available in the online repository. ARCMap (Arcus Analytica, Waterloo, Canada), was applied to re-parameterize the continuum ROM and load data prior to performing statistical operations on them through Statistical Parametric Mapping (SPM) via the MATLAB package “*spm1d*” (Pataky, 2012).

## 3 Results

All four specimens completed both combined FE and LB, and combined AR and axial loading testing at both laboratories. Specimens underwent testing on the six-axis robotic arm first, followed by the uniaxial system. FE and LB testing was conducted before AR and axial loading testing in both labs. Hotelling t-tests (p-value of 0.05) were performed between the laboratories using ARCMap on the moment-rotation behaviors for the individual loading planes. Data were determined to be normal, and no significant differences were found (all p > 0.05).

### 3.1 Combined flexion-extension and lateral bending

**FIGURE 9:**
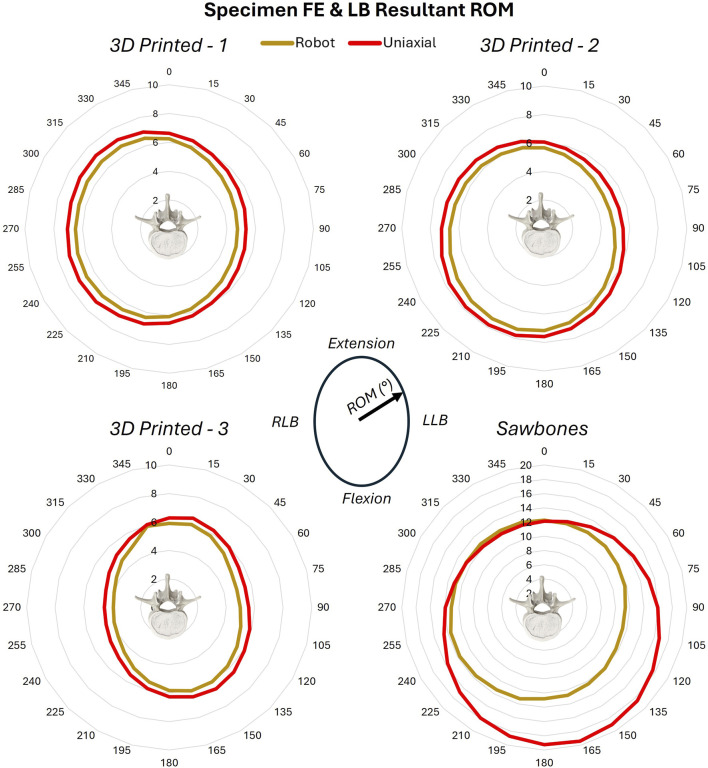
FE and LB ROM across all specimens.

In the FE and LB combined loading, resultant ROM measures around the boundaries for the 3D printed surrogates ranged from 3.9° to 7.8° across testing systems, producing a nearly circular boundary with only slightly larger ROM along planes closer to the sagittal plane (0°–180°) than the coronal plane (90°–270°). Variation in measurements between testing systems were within 0.54° ± 0.01° for each loading plane, with the uniaxial system always producing higher ROM measurements than on the robotic arm (Figure 9). There was also minimal variation the three 3D printed surrogates within each laboratory (uniaxial = 0.51° ± 0.62°, robot = 0.49° ± 0.61°) across all loading planes. The Sawbones specimen produced ROM measures ∼2.5 times larger (11.4°–19.4°) than those of the 3D printed surrogates. Variability between labs was greater for the Sawbones at 2.77° ± 2.64°; the specimen itself may account for more of this variability than the different testing systems or method of load application.

**FIGURE 10:**
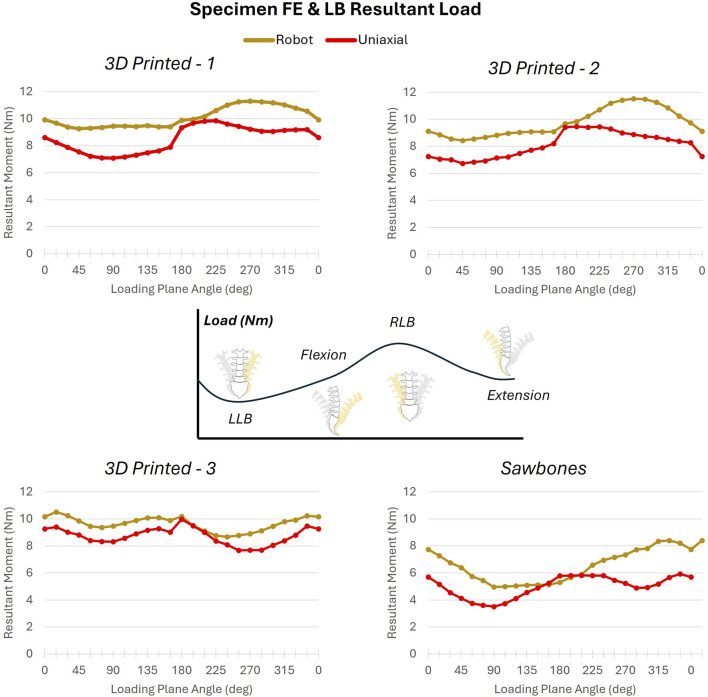
FE and LB peak resultant load across all specimens.

The rays trajectories were designed from a FE and LB combined boundary with a resultant moment of 7.5 Nm. Resultant loads in response to the rays trajectory were therefore expected to be approximately 7.5 Nm. However, for the 3D printed surrogates, resultant loads ranged from 6.74 Nm to 11.52 Nm between the two labs, illustrating some variability in response to loading. Resultant loads for the Sawbones specimen were lower, ranging from 3.5 to 8.4 Nm. Loading was higher across all slices for testing on the robotic arm for all specimens. Across three of the four specimens (excluding 3D printed Surrogate 3), loads were higher for loading planes oriented on the right side of the coronal plane and the extension side of the sagittal plane across both labs (Figure 10), indicating stiffer behavior of the specimen in these regions.

The resultant load and stiffness data across all loading planes were also translated into 3D heatmaps (Supplementary Figures S1–S4), enabling visualization of the change in load and stiffness throughout the ROM in addition to behavior at the boundary. This further illustrates biomechanical differences in planar loading along planes beyond the sagittal and coronal planes.

These data were further able to be divided into heatmaps of outward/loading and inward/unloading behaviors (Supplementary Figure S5). Hysteresis area, or the difference between inward and outward behaviors, was also assessed. This area was relatively consistent across most of the 12 loading planes for the three 3D printed surrogates, with a peak around 15° (mostly Extension with a little Left LB to mostly Flexion with a little Right LB) and 165° (mostly Flexion with a little Right LB to mostly Extension with a little Left LB). Surrogate 3 deviated from Surrogates 1 and 2 between 45° (equal FE and LB) and 120° (slightly more LB than FE). Hysteresis area for the Sawbones was largely different across anatomical loading planes between the two labs.

Off-axis loads were monitored in both testing environments to assess potential parasitic loading effects. In the robotic setup, secondary forces moments remained below 5 N and 1 Nm relative to the 7.5 Nm primary boundary moment across loading planes. In the linear–torsion system, transverse shear forces remained below 5 N throughout testing in all directions, corresponding to an off-axis moment of less than 0.92 Nm. These values were small relative to the applied primary moments and did not demonstrate systematic directional bias. Accordingly, no significant increase in observed primary stiffness was attributed to off-axis loading in either platform, consistent with the absence of significant differences in moment–rotation behavior between laboratories as determined by Hotelling t-tests (p > 0.05).

### 3.2 Combined axial rotation and axial loading

In the combined AR and axial loading, maximum left and right AR for the testing boundary varied little between the three 3D printed surrogates (10.56° ± 1.28° (left) to 11.84° ± 0.20° (right)). The Sawbones specimen had a smaller AR ROM (5.8° (left) to 6.6° (right)).

**FIGURE 11:**
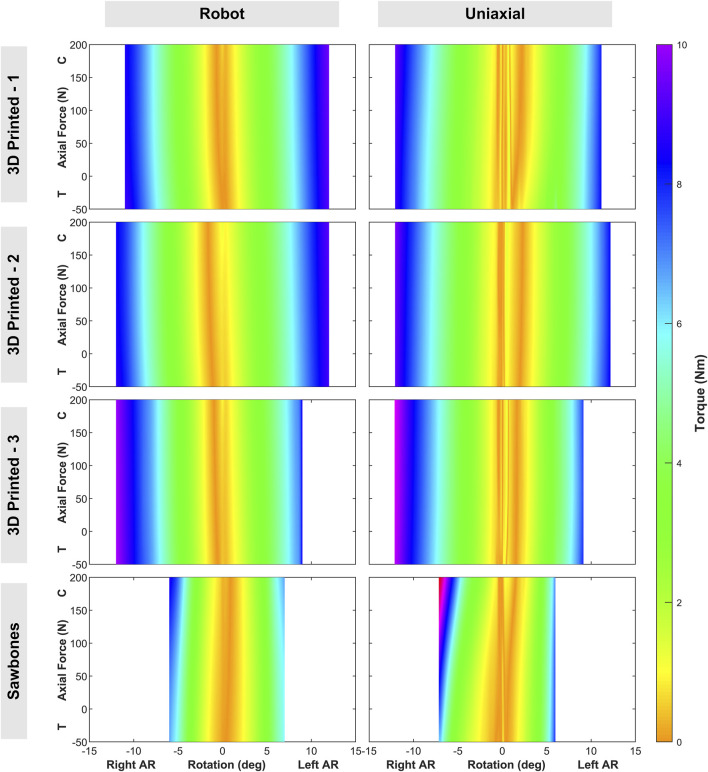
AR-Axial Loading heatmaps, by surrogate (rows) and robot (left) vs. uniaxial (right). T = Tension, C = Compression, AR = Axial Rotation.

AR moment was analyzed across the axial loading force levels (50 N in tension to 200 N in compression). As discussed previously, the relationship between planar AR loading and an axial preload has been studied with respect to AR ROM and moment at the end points of loading or inflection points marking transitions between the NZ and EZ. The data from these combined loading tests can be stitched together to map the changes on AR moment in relationship to changing AR ROM and axial loading (Figure 11). Minimal variation by level in peak load was observed for 3D printed surrogates (8.92 Nm ± 0.77 Nm (left), 9.13 Nm ± 0.97 Nm (right)). These loads were notably higher than the applied 7.5 Nm to obtain the AR ROM boundary, even at the 0 N axial loading state. A slight trend was present in only a few surrogates for either left or right AR wherein AR moment increased with increasing compression, indicating that axial (compressive or tensile) loading may not significantly influence AR ROM in these surrogates.

To further quantify agreement between testing platforms beyond visual comparison of curves, inter-laboratory absolute differences were calculated for ROM and peak load in both loading protocols across all specimens. Differences are reported as mean ± standard deviation and as a percentage of total ROM or peak load for each specimen (Table 1). For the three 3D printed (3DP) surrogates, inter-laboratory ROM differences remained below 10% in both protocols. Peak load differences were similarly small for AR + axial loading (<5% for all surrogates) and below 20% for FE + LB. Larger differences were observed for the Sawbones specimen, consistent with its greater overall variability.

**TABLE 1:** Inter-laboratory differences observed in each specimen in both protocols. Absolute differences followed by difference as a percentage of total ROM/Load in parentheses. Differences greater than 10% are highlighted in orange.

ROM (deg.)	3DP-1	3DP-2	3DP-3	Sawbones
FE + LB	0.55 ± 0.07 (8.88%)	0.54 ± 0.08 (8.56%)	0.53 ± 0.11 (9.77%)	2.90 ± 2.44 (20.88%)
AR + Axial load	0.80 ± 0.02 (3.50%)	0.83 ± 0.02 (3.50%)	0.85 ± 0.06 (4.09%)	1.21 ± 0.86 (9.81%)

Peak Load (Nm)	3DP-1	3DP-2	3DP-3	Sawbones
FE + LB	1.62 ± 0.60 (17.38%)	1.64 ± 0.64 (18.36%)	0.92 ± 0.36 (9.98%)	1.55 ± 0.95 (27.23%)
AR + Axial load	0.67 ± 0.07 (3.74%)	0.21 ± 0.08 (1.18%)	0.89 ± 0.13 (4.89%)	3.69 ± 1.53 (24.22%)

## 4 Discussion

This novel study introduces a new paradigm of multiplanar experimental testing for the spine and also presents a methodology for conducting such testing on in both robotic and load frame-based testing environments. Other studies have conducted multiplanar testing with combined FE and LB, as well as AR pure moment loads (Daniels et al., 2012; Spenciner et al., 2006) and have looked at relationships between axial loading and pure moment loading (Sun et al., 2022; Cripton et al., 2000; Bell et al., 2018; Cai et al., 2020; Patwardhan et al., 2003; Zirbel et al., 2013), particularly axial loading as a “preload” prior to experimental testing or a follower load throughout a multi-segment spinal specimen. Yet few existing studies (Holsgrove et al., 2018) evaluate equivalence in performance between different spine testing systems. This type of work is important to the field, as it improves the ability of spinal researchers to compare results across studies and to work towards more standardized spinal testing.

In general, the loading protocol was well-controlled for replicability between the two laboratories, with each lab maintaining a <2% RMS error in their control signals. All testing was conducted at room temperature. Testing order within individual specimens, wherein combined FE and LB loading, followed by AR and axial loading, was maintained. Other applied loading due to preconditioning or troubleshooting was aimed to be minimized within a testing session to control effects of these confounding factors. Determination of the combined FE and LB boundary and AR boundary prior to testing enabled creation of repeatable planar loading trajectories within the specimens’ physiological ROM for application across both testing systems. The trajectories were able to both be run continuously on a six-axis robotic arm as well as being decomposed for application on a loading frame capable of linear and rotary load application. This enabled recreation of 3D heatmap characterizations of spinal behavior in combined loadings, providing a full-field understanding of spinal biomechanics.

The requirement to define specimen-specific ROM boundaries on the robotic platform prior to application on the uniaxial system highlights an important consideration for broader adoption (Figure 8). In the present study, robotic boundary characterization was performed to establish safe physiological operating limits for the custom-manufactured 3D printed surrogates, for which no manufacturer-provided ROM specifications were available, unlike the Sawbones specimen. While the robotic system enables efficient identification of continuous multiplanar boundaries, it is not intended to be a permanent prerequisite for inter-laboratory implementation. Once boundary trajectories are characterized, whether through robotic mapping or manufacturer-provided limits, they may be shared across laboratories as standardized reference maps. A laboratory equipped solely with a linear–torsion platform could then reconstruct a down-sampled boundary by connecting end-range ROM values across rays and iteratively refining the trajectory within physiological limits. Thus, the use of robotic instrumentation in this study reflects a limitation of the 3D printed specimens rather than the proposed protocol itself.

Variability in loads from the applied 7.5 Nm boundary was noted in both combined FE and LB loading as well as AR and axial loading. Moment-controlled loading was utilized to locate these boundaries, while hybrid-control was used for applied the subsequent combined loading trajectories. The alternation between loading methodologies may have contributed to some of this variability, by introduction of coupled loading present due to uncontrolled DOF (Goel et al., 1995).

Despite actions taken for reproducibility, combined FE and LB resultant loads were markedly higher for testing on all samples on the robotic arm and a larger ROM for uniaxial testing system. The specimens were noted to have mechanically changed, especially the Sawbones sample, between the testing laboratories. Delamination damage was observed on the Sawbones sample between the L2-L3 level due to accrual of degradation from prior testing on the specimen, perhaps also contributing to the presence of bilateral asymmetries in LB and AR. Furthermore, though specimens were stored in UV protectant bags and obscured from overhead lights while not being tested and while being transported between the two testing laboratories, exposure to UV lighting and airport X-ray security scanning during transit may have impacted the materials present in these photo-polymeric surrogates. Additionally, friction within the passive XY slide of the linear–torsion system may have introduced minor resistive forces during coupled motion and load reversals, potentially contributing to hysteresis differences. These XY frictional forces could not be quantified with the current bi-axial load cell in the linear-torsion system. Transverse shear forces (Z), however, remained below 5 N (<0.92 Nm off-axis orthogonal moment) during all the tests.

The combined FE and LB loading highlighted additional asymmetries in the 3D printed surrogates, as well. The 3D printed surrogates were derived from real patient anatomy which maintained any patient-specific spinal asymmetries. Unlike pure moment testing alone, loading along the additional loading planes every 15° informs asymmetries that appear in spinal movements that are not exclusively along the sagittal or coronal planes. This helps to capture the reduced left LB in this anatomy, which impacts not only pure left LB but combinations of bending along the sagittal plane in tandem with left LB.

This is also illustrated by the differences in loading along planes between 45° (equal FE and LB) and 120° (slightly more LB than FE), particularly for 3D printed surrogate #3. Surrogate #3 illustrates a similar left LB boundary to surrogates #1 and #2, but a reduced right LB edge to the boundary. Though this resulted in more equivalent resultant load values across all loading planes, it likely illustrates some asymmetries introduced into the surrogate’s behavior due to the fabrication process. Though the primary objective of this work was to compare results of a novel testing methodology between two distinct testing systems, this work may also be used to guide areas of further refinement of these spinal surrogate models.

While numerous studies have established that lumbar FE behavior is influenced by axial follower loads, this study investigated the impact of an axial follower load on AR behavior, for which literature is limited. The results from this study indicated that AR load was not significantly impacted by axial loading. The homogenous composition of the 3D printed surrogate and Sawbones surrogate IVDs may have contributed to this phenomenon, as cadaveric lumbar IVDs have a heterogeneous structure in which the annulus fibrosus within the IVD adapts to increasing axial load to protect the nucleus pulposus (Nie et al., 2024). Future cadaveric lumbar spine testing should be conducted to verify this hypothesis.

The small sample size (n = 4 surrogates) limits the statistical power of this study and restricts the generalizability of the findings. Only a single commercially available Sawbones specimen was tested due to limited availability and subsequent market discontinuation, which precluded intra-specimen comparisons for that analogue. Although multivariate statistical comparisons did not demonstrate significant inter-laboratory differences, the study may be underpowered to detect subtle effects, particularly specimen-specific asymmetries or small stiffness variations. As such, the results should be interpreted as preliminary validation of methodological equivalence. Future investigations should include a larger number of specimens. Nevertheless, the results from this study provide a vast pool of data from which previously undocumented aspects of spinal behavior can be analyzed. An additional structural limitation is that the anterior and posterior longitudinal ligaments (ALL and PLL) were not explicitly modeled as discrete structures in the 3D printed surrogates (Figure 1). Segmental stiffness was instead achieved through the composite material properties of the intervertebral disc region and surrounding printed structures, representing an anatomical simplification. As the ALL is a primary stabilizer in extension, its absence as a distinct structure may influence extension-dominant mechanics. Incorporation of anatomically discrete ligamentous elements remains an area of improvement in these 3D printed models.

## 5 Conclusion

This first-of-its-kind inter-laboratory study validated two novel combined-loading protocols, characterizing the multiple dimensions of spinal behavior, on two commonly used biomechanical testing systems. Both protocols were able to be decomposed and translated from a six-axis robotic arm to a two-axis linear-torsion testing system, lending credibility to this method as a standardizable, reproducible process among biomechanics laboratories. Spinal surrogate models aided this work by providing a repeatable, transportable, and inexpensive medium for testing between laboratories located over 1,400 km apart in a span of 2 months. The results of this work demonstrate a strong agreement in spinal ROM and resultant load across the two laboratory setups, despite numerous possible sources of variability. Furthermore, both laboratories were able to utilize the methods to highlight specimen asymmetries and biomechanical responses that would be otherwise overlooked by traditional pure moment testing. This work lays a foundation for future multidimensional testing for capturing complex biomechanical behavior of the spine.

## Data Availability

The datasets presented in this study can be found in online repositories. The names of the repository/repositories and accession number(s) can be found in the article/Supplementary Material.
