# Supplementary material for: An inter-laboratory study of the multi-dimensional behaviors of analogue lumbar spine surrogates: towards standardization in spine testing
Source: Front Bioeng Biotechnol. 2026 Apr 9;14:1769107. doi: 10.3389/fbioe.2026.1769107 (PMC13102567; doi:10.3389/fbioe.2026.1769107)
Supplement: Supplementary file 1 [file DataSheet1.pdf]

*Supplementary Material*

## 1 Supplementary Data

The data and the analysis scripts associated with the manuscript "*An Inter-laboratory Study of the Multi-dimensional Behaviors of Analogue Lumbar Spine Surrogates: Towards Standardization in Spine Testing*" are provided in the McGill-Wake GitHub repository and can be accessed at https://github.com/siril-teja/McGill-Wake.git. For any other reasonable requests on additional materials and resources, please contact the authors.

## 2 Supplementary Figures

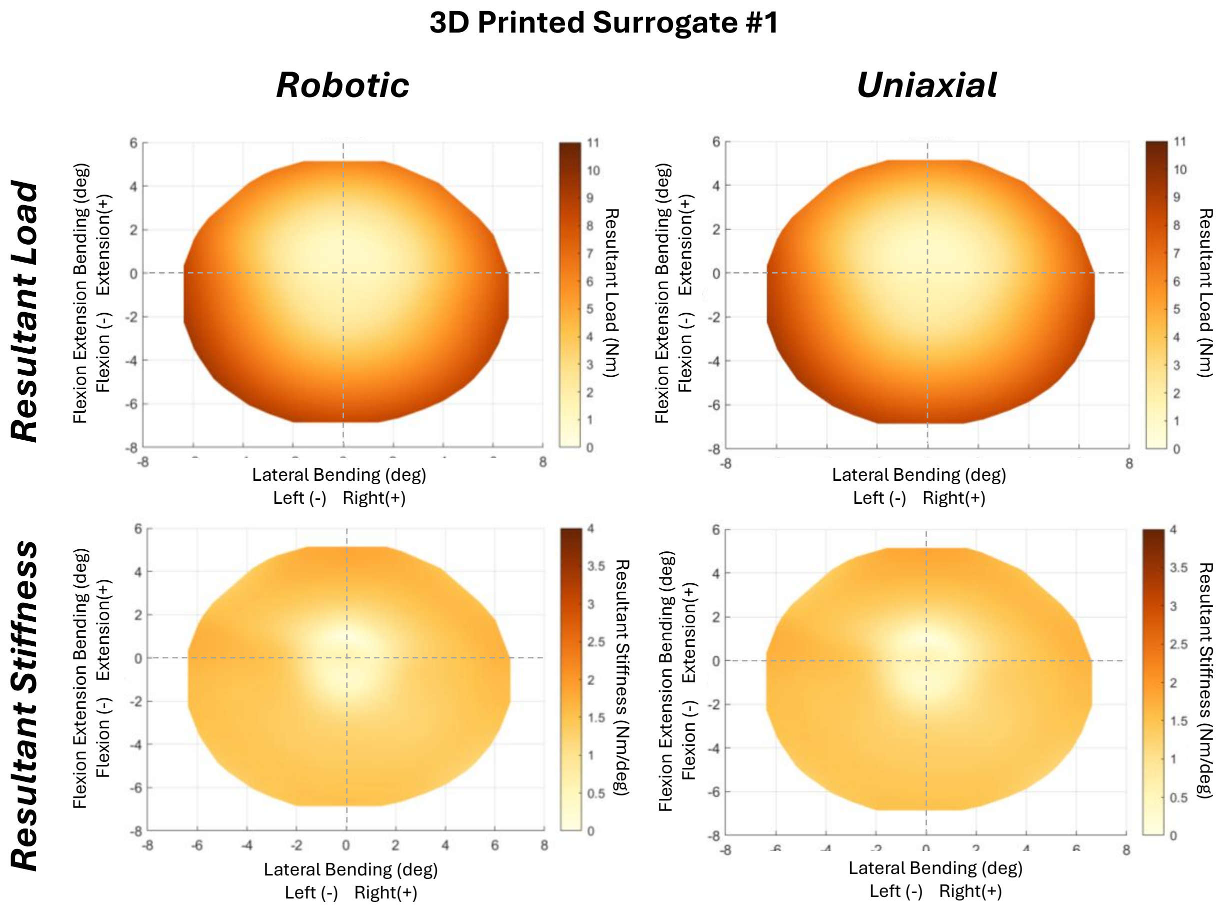


**Supplementary Figure 1.** Resultant load (top) and stiffness (bottom) heatmaps for robotic (left) and uniaxial (right) testing systems for 3D Printed Surrogate #1.

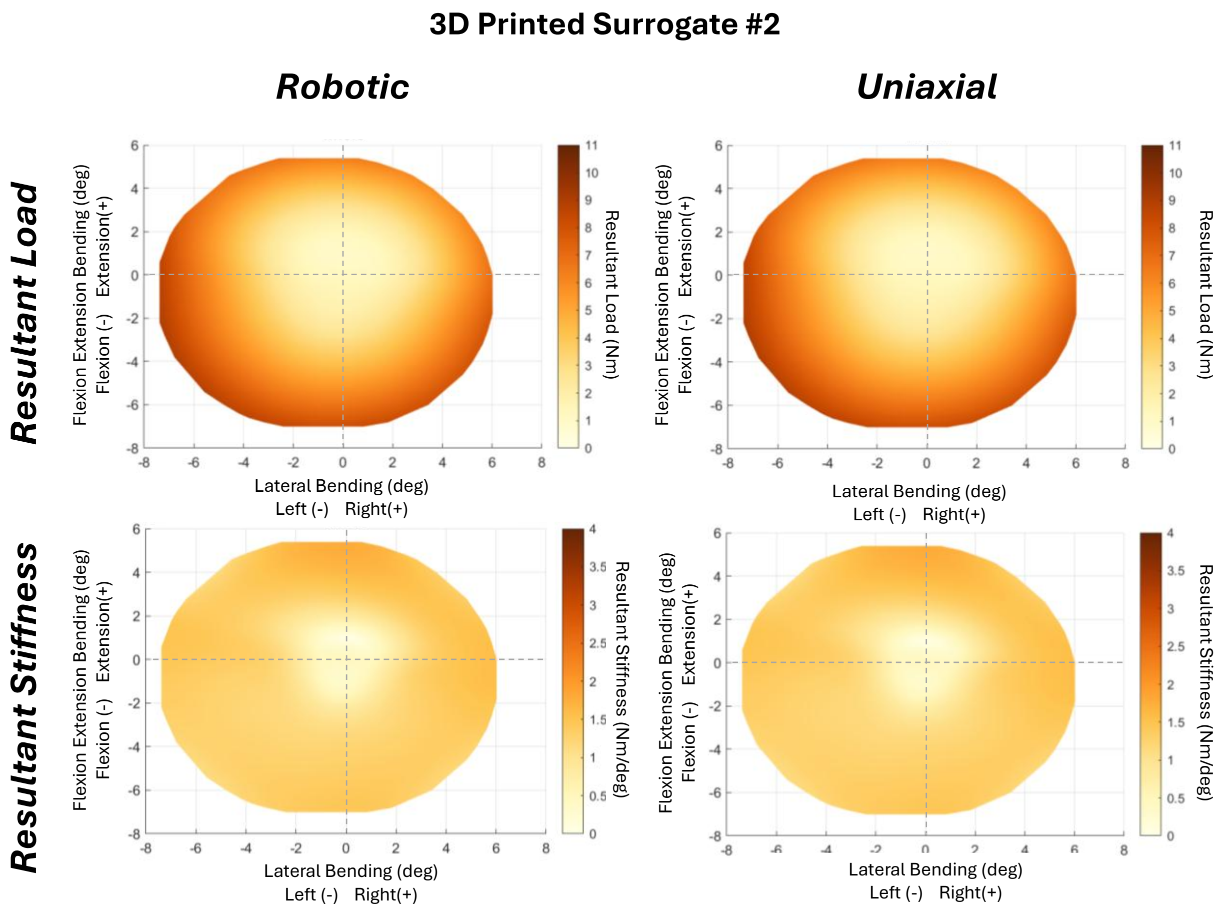


**Supplementary Figure 2.** Resultant load (top) and stiffness (bottom) heatmaps for robotic (left) and uniaxial (right) testing systems for 3D Printed Surrogate #2.

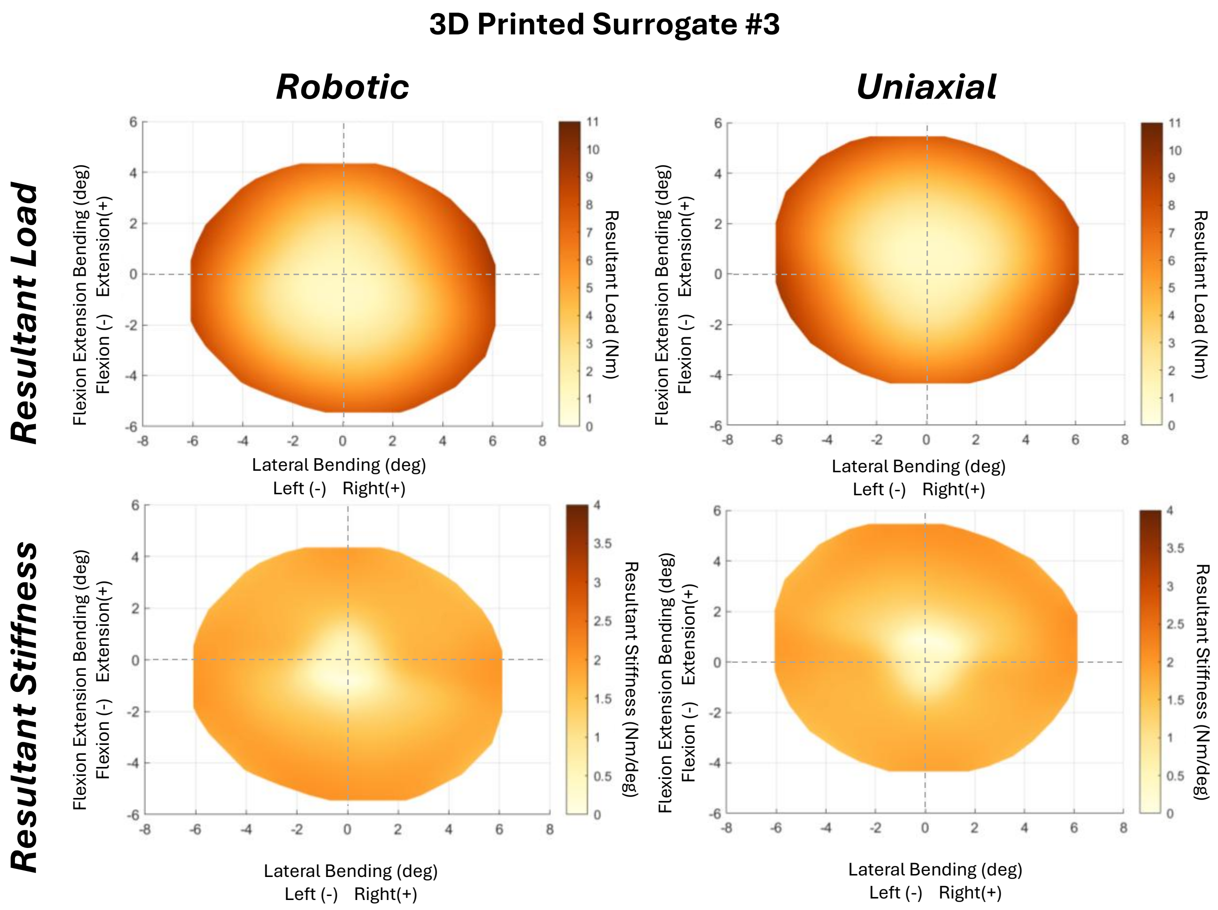


**Supplementary Figure 3.** Resultant load (top) and stiffness (bottom) heatmaps for robotic (left) and uniaxial (right) testing systems for 3D Printed Surrogate #3.

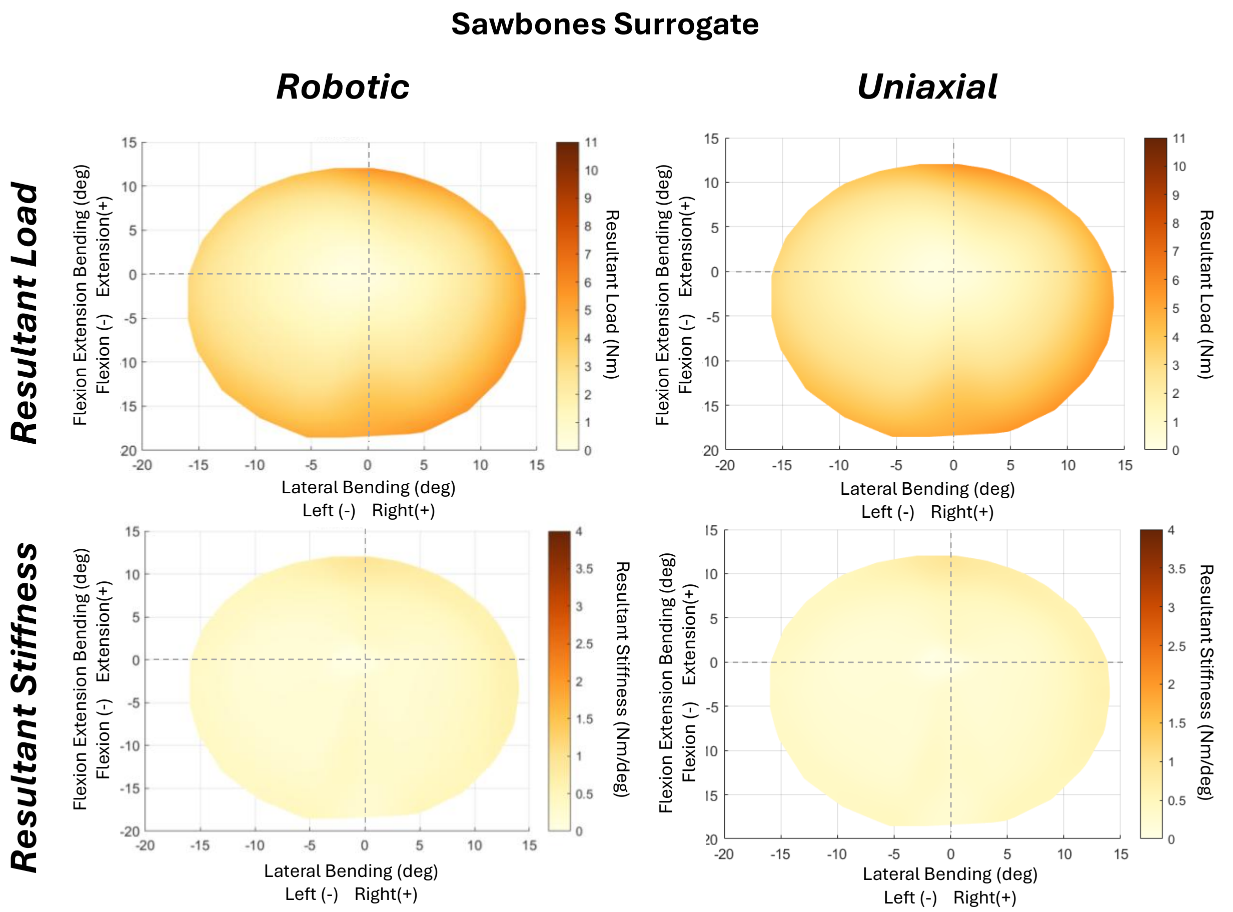


**Supplementary Figure 4.** Resultant load (top) and stiffness (bottom) heatmaps for robotic (left) and uniaxial (right) testing systems for Sawbones Surrogate.

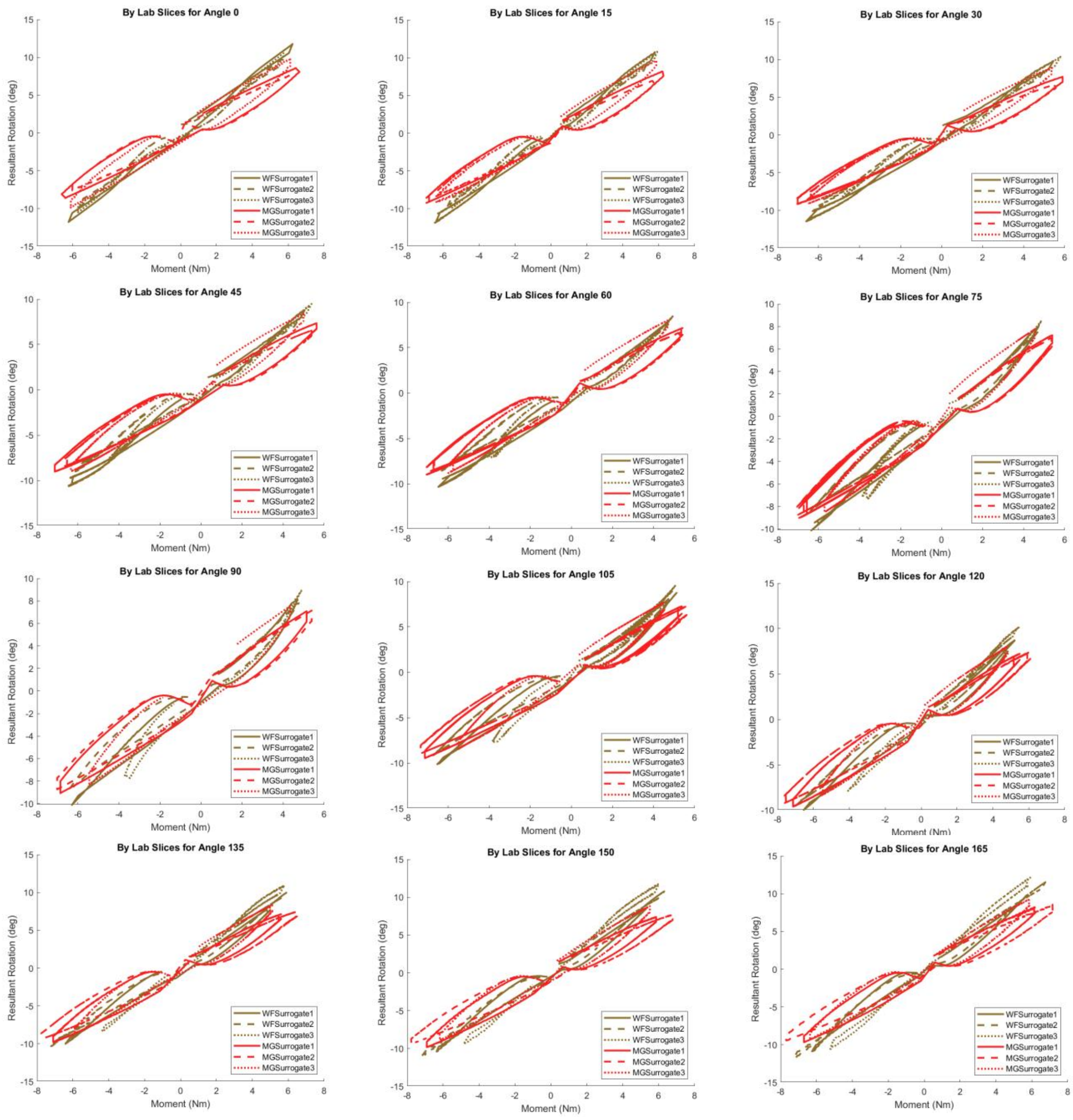


**Supplementary Figure 5.** Resultant load-displacement (moment-rotation) plots for each loading plane of the 3D printed surrogates. WF = Wake-Forest lab, MG = McGill Lab.
